# Role of Technology in Self-Assessment and Feedback Among Hospitalist Physicians: Semistructured Interviews and Thematic Analysis

**DOI:** 10.2196/23299

**Published:** 2020-11-03

**Authors:** Andrew Lukas Yin, Pargol Gheissari, Inna Wanyin Lin, Michael Sobolev, John P Pollak, Curtis Cole, Deborah Estrin

**Affiliations:** 1 Medical College Weill Cornell Medicine New York, NY United States; 2 Cornell Tech New York, NY United States; 3 Feinstein Institutes for Medical Research Northwell Health Manhasset, NY United States; 4 Department of Medicine Weill Cornell Medicine New York, NY United States; 5 Department of Population Health Sciences Weill Cornell Medicine New York, NY United States

**Keywords:** feedback, self-assessment, self-learning, hospitalist, electronic medical record, digital health, assessment, learning

## Abstract

**Background:**

Lifelong learning is embedded in the culture of medicine, but there are limited tools currently available for many clinicians, including hospitalists, to help improve their own practice. Although there are requirements for continuing medical education, resources for learning new clinical guidelines, and developing fields aimed at facilitating peer-to-peer feedback, there is a gap in the availability of tools that enable clinicians to learn based on their own patients and clinical decisions.

**Objective:**

The aim of this study was to explore the technologies or modifications to existing systems that could be used to benefit hospitalist physicians in pursuing self-assessment and improvement by understanding physicians’ current practices and their reactions to proposed possibilities.

**Methods:**

Semistructured interviews were conducted in two separate stages with analysis performed after each stage. In the first stage, interviews (N=12) were conducted to understand the ways in which hospitalist physicians are currently gathering feedback and assessing their practice. A thematic analysis of these interviews informed the prototype used to elicit responses in the second stage.

**Results:**

Clinicians actively look for feedback that they can apply to their practice, with the majority of the feedback obtained through self-assessment. The following three themes surrounding this aspect were identified in the first round of semistructured interviews: collaboration, self-reliance, and uncertainty, each with three related subthemes. Using a wireframe, the second round of interviews led to identifying the features that are currently challenging to use or could be made available with technology.

**Conclusions:**

Based on each theme and subtheme, we provide targeted recommendations for use by relevant stakeholders such as institutions, clinicians, and technologists. Most hospitalist self-assessments occur on a rolling basis, specifically using data in electronic medical records as their primary source. Specific objective data points or subjective patient relationships lead clinicians to review their patient cases and to assess their own performance. However, current systems are not built for these analyses or for clinicians to perform self-assessment, making this a burdensome and incomplete process. Building a platform that focuses on providing and curating the information used for self-assessment could help physicians make more accurately informed changes to their own clinical practice and decision-making.

## Introduction

As we explore a world where machine learning and advanced algorithms are increasingly used to assist in medical care, consideration of how to empower individuals to use these data themselves is often neglected. The aim of this project was to explore which tools or modifications to existing systems could most benefit hospitalist physicians, matching both their intrinsic and professional desires for self-assessment and improvement. Although applicable to many clinicians, hospitalist physicians represent a group that is specifically challenged in obtaining such feedback, as they do not see the patients they treat after discharge. Accordingly, they lack the opportunity to adjust or realize the benefits and shortcomings of the care provided. As a team of technologists and clinicians, we set out to understand how hospitalist physicians seek feedback to improve their practice, and to explore potential technical solutions that might be able to support them using a qualitative approach grounded in an iterative design process.

Embedded in the culture of medicine, physicians embrace the value of lifelong learning [1]. The pursuit of this goal is engrained in medical trainees, particularly in the form of self-directed learning and self-assessment [2], and is reinforced in the Physician Competency Reference Set produced by the American Association of Medical Colleges [3], the Common Program Requirements for residents by the Accreditation Council for Graduate Medical Education [4], and the requirements to participate in continuing medical education throughout a clinical career [5]. Nevertheless, it is recognized that self-assessment is a flawed, challenging process that in the wrong environment can adversely affect the participating clinicians. Physicians have a limited ability to accurately assess themselves, resulting in a feeling of imposter syndrome that can further reduce the physician’s trust in other forms of self-assessment [6-8].

As a result, self-directed learning and self-assessment are commonly complemented by other models aimed to support the growth of the physician. One favored model is audit and feedback, a process that aims to show an individual whether or not performance is on par with a desirable target metric. This model has led to various behavior changes in physicians, including improving antimicrobial stewardship, completing relevant tasks such as discharge summaries, and helping inform appropriate use of tests and screens [9-12]. However, even with clear target metrics, the effectiveness of this method depends on baseline performance, how the feedback gets delivered, and the clarity of the action plan [13-15]. A second method is peer-to-peer or observation-based assessment, which ideally involves direct observation of the feedback receiver by the feedback giver, an appropriate institutional culture, and appropriate delivery of the feedback [16]. Thoroughly studied in medical training, peer-to-peer assessment has strengths in consistency and predictability of long-term quality, but greatly depends on trust, time, and attention to confidentiality [17-22]. Third, physicians can use information from patient feedback surveys to guide learning, but studies have shown that there are many contextual factors that influence actionable change. These include an appropriate work culture that embraces use of patient feedback, credibility of collection, and specific data content [23-25]. Other studies have unfortunately shown that physicians have at best a mixed view of patient feedback if they use it at all, with a negative view being more likely due to distrust of credibility, use to threaten their jobs, or distrust of the administrative motivations for using this feedback [26-28].

This study is based on the recognition that although there has been substantial research focusing on helping physicians to improve, progress is still needed to gain a deeper understanding about the feedback physicians currently use and the type of feedback they prefer [29,30]. In addition, although electronic medical record systems have been studied in the context of quality improvement [31,32], there appears to be a lack of tools or research described in the literature aimed at using existing technology and data available to support individual physician improvement.

## Methods

### Participants

Hospitalist medicine physicians from a single, large academic medical institution were invited to participate in this study. Randomly selected members in the department were emailed with information about the study to ask for their participation and were offered the option to speak in person, over the phone, or over video conferencing platforms. A total of 44 individuals were emailed with 17 responses obtained, 5 of whom were not interviewed due to scheduling limitations or loss in follow-up communications. Twelve individuals met our criteria and agreed to participate in the first round of interviews. A randomly selected subset of 4 participants were then interviewed again in the second round of interviews. All participants provided informed, written consent for their participation in the study and were not compensated for their participation. This study received an exempt status from the Cornell University Institutional Review Board under protocol number 1912009284.

### Demographics

Among our interviewees, the average total years of work experience as a hospitalist was 5.8 years with the average time working at the current institution being 3.9 years. Five of the twelve participants were female. Ten of the participants described teaching as one of the main activities in their current role.

### Data Collection: Semistructured Interviews Round 1

Semistructured interviews were used for a thorough examination of each participant’s experience and motivations. Our interview methods closely mirrored those described previously [33,34]. Interviews were conducted in a private space either in person, over the phone, or over video conferencing platforms depending on the participant’s preference and availability. Each interview lasted about 45 minutes and was composed of a single study participant and two members from the research team. All interviews were conducted by AY, PG, and IL to provide consistency across interviews. One team member asked questions while the other took notes on any observable body language, tone of response, and potentially overlooked content. The interviewer used a set of predetermined open-ended questions developed following a previously described framework to understand all ways that participants pursue improvement in their work, seeking to understand how they get feedback, how they seek to improve, and what information they wish they could obtain [35]. This interview technique allowed for participants to dive deeply into their experiences and speak openly about them. All interviews were recorded and transcribed by members of the research team and combined with the notes taken during the interview itself. AY, PG, and IL reviewed each audio file and transcript for consistency and accuracy.

### Data Analysis

A thematic analysis was performed following the methods of Braun and Clarke [36] to analyze the data owing to its strengths of accessibility and flexibility in analysis, enabling deep exploration of a rich dataset and to identify repeated concepts. The thematic analysis covered six phases: (1) familiarizing, transcribing, and reviewing all recordings and notes, and rereading each transcript prior to proceeding; (2) generating initial codes individually to allow for diversity in perspective and then discussing collaboratively to combine and rework codes; (3) searching for themes and subthemes that emerged as consistent patterns in coded elements; (4) reviewing potential themes and subthemes in relation to the dataset to refine themes and codes; (5) defining and naming themes and subthemes to best express the final concepts captured; and (6) producing a report [36]. After individual coding of two transcripts, the team developed a set of unique codes used for the remaining transcripts. AY, PG, and IL met to revise this codebook every two transcripts. Saturation, the point at which no new codes were needed, was reached after the 10th transcript. After completing the coding of all interview transcripts, the codes were consolidated into 22 categories by consensus, with final consolidation through group discussion leading to the final unifying themes and subthemes.

### Wireframe Development

Using the identified themes and subthemes, the research team developed a wireframe of an app aimed at addressing user needs. Development of the wireframe involved an interdisciplinary team of technologists (DE, JP, PG, IL), clinicians (CC, AY), and a behavioral scientist (MS) to create a wireframe that addressed the identified user needs. This wireframe was then used for the second round of interviews.

### Data Collection: Round 2 of Semistructured Interviews With the Wireframe

The research team set out to assess how well the wireframe addressed the themes and subthemes using semistructured interviews [33,34]. These interviews occurred with a subset of the original interview participants, allowing interviews to more quickly delve into more substantive topics. Each interview lasted about 30 minutes. The researchers showed each screen of the wireframe, using a script to give a basic overview followed by a set of predetermined open-ended questions [35]. Interviewees were shown the wireframes through video conference screen share or on one of the researcher’s computers. All interviews were recorded and transcribed. Analysis focused on determining how the prototype addressed the themes and subthemes.

## Results

### Thematic Analysis of First Round Interviews

The research team identified themes and subthemes related to self-directed assessment and learning. The three themes that emerged were: collaboration, self-reliance, and uncertainty. Within each theme, three subthemes were extracted from the interviews with participants (Table 1).

**Table 1:** Descriptions of themes and subthemes.

Themes and subthemes		Description
**Theme I: Collaboration**		
	I.I: Physicians are looking for feedback	Whether new or seasoned, physicians use many currently available resources to gather feedback for themselves but have difficulty sharing feedback with one another and are still looking for more for themselves.
	I.II: Physicians have specific people they consult for feedback	In reviewing past decisions, physicians defer to a close ring of current or past colleagues or family members in the medical field rather than purposefully finding external experts or people they are less comfortable with.
	I.III: Physicians interpret feedback more negatively than likely intended	Although feedback is usually not intended to do so, physicians tend to have a strong, negative emotional response to the feedback they receive.
**Theme II: Self-Reliance**		
	II.I: Physicians have go-to resources for learning	Participants have an array of resources they readily rely on for learning, with little variation among participants.
	II.II: Physicians build workarounds	Physicians build and maintain workarounds to obtain information of interest, but they find them to be inefficient and time-consuming.
	II.III: Medicine can feel like a solo sport	Although the practice of medicine is commonly thought of as a team environment, participants sometimes feel they are acting on their own, which can be challenging.
**Theme III: Uncertainty**		
	III.I: Physicians like numbers but need more context	With the current data available, physicians find it challenging to create actionable learning points as the data usually do not capture enough context.
	III.II: Physicians cannot always find the “right” answer	There may not be a “right” answer in medicine much of the time, and physicians have a hard time finding out if their actions in the past were the best actions given the situation at the time.
	III.III: Physicians’ actions are limited by uncertainty about workplace cultural expectations	Uncertainty with workplace cultural or collaborative expectations creates friction for them in giving feedback.

#### Theme 1: Collaboration

Physicians rely on one another for feedback and point-of-care advice. With respect to feedback from colleagues, three subthemes arose. First, physicians do not receive much feedback in their daily work even when looking for it. Second, when they do solicit feedback, they typically only go to the people they trust. Last, on the occasions that physicians do receive feedback from one another, they tend to interpret the feedback more negatively than it is likely intended.

##### Subtheme 1.1: Physicians Are Looking for Feedback

Most of the respondents mentioned a lack of feedback in their daily work, a trend observed among both new and more experienced physicians. Some participants reflected on having more established learning and feedback mechanisms in residency. As one of the interviewees said: “there's not enough feedback in our careers, as soon as you finish medical school and residency and then you just go out into the world and, uh, basically get no feedback unless you look for it.” [participant 3]

As a result, physicians are trying to get this feedback.

When I do my signout and email my signout to the next provider I will usually say if there is anything that is blatant or if you are noticing something that you want to comment on or give me feedback on then please do because there aren’t many other ways for hospitalists to get feedback on their clinical judgment.

participant 5

When asked about giving feedback to other physicians, an interviewee mentioned that they have trouble giving direct constructive feedback: “I try to give some feedback very sneakily just by trying to give an update… by telling them how that [patient] is doing clinically, which I think implies some feedback.” [participant 8]

##### Subtheme 1.2: Physicians Have Specific People They Consult for Feedback

Although a physician may ask for second opinions from experts or colleagues during real-time point of care, they tend to only go to people they trust when reflecting on whether a previous diagnosis could have been different. Interviewees mentioned these inner circles being friends from medical school, trusted colleagues, or family members in the medical field: “I definitely look to some of my most trusted colleagues to try to debrief and go over if I really did mess up, could have done something different, what would I do next time, what would you have done, etc.” [participant 12]

##### Subtheme 1.3: Physicians Interpret Feedback More Negatively Than Likely Intended

On the occasions that physicians receive feedback, they share a tendency to feel negatively about that feedback. Although it is unlikely that the intention of the feedback giver was to create these negative emotions, participants have a notably negatively biased emotional response, even about items that were not necessarily their fault. One participant described this as:

I think most people, if you tell them “Hey, you could work on X, Y, Z things,” and I see this when I talk to residents and students and have felt this myself in that position, that you feel a little personally attacked.

participant 3

Others described this emotion as pervasive and strong. After hearing some feedback, one participant said:

I felt so awful, and the thing is I didn’t even know the patient was in this situation. But, I think it made me feel so bad that it heightens your awareness after something that like...you’re so scared and don’t wanna repeat your mistakes so you overcompensate.

participant 6

These feelings extend beyond negative feedback alone. Even in receiving positive feedback, participants expressed having a hard time feeling genuinely good if the feedback was not constructive or clear. One participant said:

It's not all positive feedback that I find useless but it’s that very generic like obviously not very thoughtful feedback that I find useless. And then I can’t even feel good about it because I don’t even know what I did to deserve that comment. I feel like I don’t even own that comment.

participant 5

#### Theme 2: Self-Reliance

Participants shared that when it comes to taking actions and making decisions in challenging settings, they often rely on their own resources, skills, logic, and experience. Three main self-reliance trends were observed: using go-to resources, building workarounds, and managing the lonely nature of the work.

##### Subtheme 2.1: Physicians Have Go-To Resources for Learning

Almost all participants shared a list of literature resources that they heavily rely on when it comes to learning and decision-making, including UpToDate, journals such as *New England Journal of Medicine* and *Journal of the American Medical Association*, and even social platforms such as Twitter. One participant described:

I use UpToDate the most in my clinical work to help me work through an answer or read a little more of background about treatment… I use Twitter to passively scroll for new ideas or new papers, but I will save a tweet if I thought it was especially helpful that I could look at it later.

participant 12

In addition, physicians are reviewing their past patients in the medical record, as one participant described:

After we get off service we tend to stalk the list and read about them and what our colleagues did. If there’s a patient that I want to follow long-term you can make your own list on the electronic medical record and keep track of them that way. You can see what happens in the long term.

participant 6

##### Subtheme 2.2: Physicians Build Workarounds

Although they have some resources, many participants stated that they create additional workarounds to find or keep relevant information. Some create lists of patients to track and revisit, while others keep documents with facts that they have learned from different sources. They expressed that these self-made systems take a lot of time and energy to maintain. One participant mentioned that:

I have an ad-hoc system which is not easy to do. Essentially, after completing every block, I compile a list of patients on that block, then I manually enter each patient ID into the electronic medical record. It’s not easy to track patients, so I have to move them over, one by one. Then I have a set up where I can see their last admission date and their last outpatient date. I keep tabs on patients that are still active or that I want to keep an eye on...it’s helpful to see what happens when they get discharged; was my judgment ok, did they come back. It’s helpful to see how our doctors synthesize their problem, and if they get readmitted. That is normally a flag for me, I want to see whether they were readmitted because I could’ve done something differently, I want to know about and see what I can do in the future. Sometimes their disease progresses and I can’t do anything about it, but it is also helpful to know.

participant 10

Another participant described:

I have a long and incredibly messy running document on Evernote that’s just quick pearls or facts that I’ve just learned along the way and I type into that every once in a while, either on my phone or on my computer. It’s just saved there, and I don’t do a very good job of organizing it or improving the layout of it. I will just search for a specific word and find it in the document, but it could certainly be much more elegant.

participant 8

Although some participants have been able to continue their workarounds, some have given up along the way as things can quickly become too hard to keep up with or keep organized:

I never really looked at my ongoing notes again. It just took way too much time and it’s something that I never looked at again. I think it points more towards organization; I need to be more organized in terms of where to store things so that I can retrieve it easily...I tried doing it on the computer with folders like a folder of evidence, physical diagnosis, or by disease. I have resources for cirrhosis, heart failure, and pneumonia and all these things, I have even aligned them with the competencies of hospital medicine. I even tried different things; I definitely need to be excellent in organizing my folders, but you just never go back to it.

participant 7

##### Subtheme 2.3: Medicine Can Feel Like a Solo Sport

Although the practice of medicine is commonly thought of as a team environment, participants expressed that at times they feel a strong burden that they must do certain things on their own—a burden that they can find challenging. Some noted this feeling in relation to the need to monitor their own performance. One participant noted that:

If a colleague takes over a patient and totally changes my plan and ends up being right about new management, there is no way for me to know about that without me looking. If I admit somebody, and I say that they have pneumonia and they ended up having a PE [pulmonary embolism], basically nobody tells me that I was wrong about that, which is insane.

participant 3

Another remarked:

When it comes to my clinical decision making, I think I have taken responsibility for feedback upon myself. When I go off service, for at least the next 2 or 3 days afterwards I pop into the charts of the patients that I've passed off and see what happened.

participant 9

Others noted a similar feeling related to staying current on advances in medical knowledge:

I think there are moments where I wonder if my level of knowledge is as up-to-date as it ought to be. I think there are areas that I probably could look up more out of curiosity, I just haven’t.

participant 1

#### Theme 3: Uncertainty

Participants expressed that one of the most difficult aspects of assessing themselves is the uncertainty in their daily environment, making some actions and assessments challenging. This centered around three subthemes: uncertainty with data, with finding a correct answer, and with cultural and collaborative expectations.

##### Subtheme 3.1: Physicians Like Numbers but Need More Context

Almost all participants openly expressed the importance of data in driving their decisions and actions in real-time clinical work, but when it came to assessing their past performance, they expressed a lack of data that they trusted beyond rereading the clinical courses of patients. One participant explained: “the problem with feedback in hospital medicine, it’s hard to get outcomes data or change of care data because so many hospitalists are in charge of someone.” [participant 4]

In addition, participants expressed the challenge of using qualitative feedback from students and residents, as they commonly offer conflicting or unactionable comments:

they are usually very generic...so someone will say “she doesn’t teach enough” and someone else will say “she teaches too much, rounds go on too long” and so I have noticed that oftentimes the constructive feedback only brings down my mood but doesn’t really add much.

participant 5

When asked to discuss what data they would be interested in having, participants openly expressed interest in data about readmissions, length of stay, and mortality, but were quick to mention reservations. One participant described the challenge with variance in the data:

I would love to know in general what of the patients I see are more or less likely to be readmitted compared to my peers or compared to some average, but the problem is, for example, 6 months into this job that I would imagine there would be so much variation just by pure random chance that it would probably take at least a sample size of 1 to 2 years to be able to tell us if any of our numbers were actually reflective of true differences from the mean or whether it was just statistical wackiness.

participant 8

Another described the challenge of figuring out how to attribute data to a single person:

It can get pretty noisy particularly at the physician level because physicians hand off a lot. One of the reasons the length of stay data is an issue is if I am on service for 7 days half of the people I discharge, I wasn’t really involved with their care upfront.

participant 2

In other words, participants express difficulty finding reliable data that feels applicable, actionable, and timely. Even when discussing potentially valuable data points, participants worry that these data points do not consider the context and variability associated with each person’s practice.

##### Subtheme 3.2: Physicians Cannot Always Find the “Right” Answer

Although they have doubts about some of the data points, participants are nevertheless looking to know if they made the right decisions. Currently, they primarily do this by checking in on a patient’s record for some period of time to understand the outcome of the patient, even though this can still be challenging. One participant described:

If I look back at an old H & P [History and Physical], it may not reflect the correct diagnosis for this person because I didn’t really get a handle on how to deal with the situation until day 2 or day 3. There isn’t really a clear way as it’s currently formulated to mark where it was in the chart that you had your breakthrough and figured out what it was and figured out what you wanted to do, so it requires a lot of wading through.

participant 8

Even with the ability to look back, it can be hard to find an exact answer. Participants expressed managing this in different ways, but many acknowledged how this uncertainty is a part of their job and challenging to navigate. One participant said:

If things aren't clear later on and we don't have the data to figure out whether they will be clear, we'll just have to move on. I think dealing with uncertainty is an important part of our job...it would be paralyzing to try to make sure that for every diagnosis we made or every treatment we offered, we had an exact knowledge of whether that was right or wrong. It's just impossible to know for some of those cases.

participant 9

Another described this challenge of parsing through what to assess and having too many factors to be able to clearly delineate what might be right or wrong as:

You could do something perfectly right and it’s still difficult to know because a patient was so sick and had a bad outcome, there could be some emotional things tagged to it like if they’re young or if you didn’t get along with the patient and the family back in heightened emotions and make it difficult to. Or there is some conflict or you know you and a consultant weren’t seeing eye to eye so there are a lot of different factors.

participant 12

As a result, participants expressed different ways of managing this challenge. One explored this a step further by stating the importance of reasoning as opposed to assessing whether or not the decision was correct, stating:

I guess rather than what the ultimate answer was, it matters more if I got there for the right reason and did the appropriate workup. Unless there was a right reason to making that decision, then it doesn't feel good to make the right decision for the wrong reason and to not have the right thought process behind it.

participant 3

##### Subtheme 3.3: Physicians’ Actions Are Limited by Uncertainty About Workplace Cultural Expectations

Participants expressed that uncertainty with cultural or collaborative expectations creates friction for them in giving feedback. One remarked how normal conversations can become uncomfortable due to this issue, stating:

If there’s not a cultural expectation, then it feels like a big deal even if you want it to not be a big deal and just be casual, quick feedback. It always makes the thing feel bigger than it is.

participant 8

Another commented on how this lack of structure can create challenges for the feedback giver and discourage future willingness to share, saying that: “There is no mechanism and my experiences have been quite negative in terms of actually being able to provide feedback to my colleagues.” [participant 1]

Participants added how uncertainty with feedback structures create major stresses, even in important moments when they feel feedback to be necessary. One participant said: “It was scary because he’s a senior attending and I was in my second year, but I felt that it was something that couldn’t not be told.” [participant 12]

In another way, this lack of expectations can cause a lot of stress as they try to parse through messages thinking there might be hidden feedback. One participant shared:

I’m sure the implicit message is here are the updates and here are the considerations that should’ve taken place. I’m sure that happens, but I don’t think that’s ever explicitly stated like “I would’ve done this rather than that.” I can’t read the mind of the person delivering updates, but I do wonder if that’s what they’re trying to convey just by human nature. Are they telling me this just because or is it subliminal messaging or am I just being delusional?

participant 7

One participant described the cultural challenge in balancing this:

I have done a lot of reading about the concept of uncertainty and I think that the reality is that we deal with a lot of uncertainty clinically in our diagnoses and therapies and so on. I think that one thing that is unfortunate is that there is no way to really discuss that uncertainty with other people and I think that often we’re not honest about the uncertainty that we are experiencing because it’s not perceived to be—you’re not a very good doctor if you actually are not able to manage that or appear confident despite the uncertainty.

participant 1

### Second Round Interviews With a Wireframe

Based on the thematic analysis, a wireframe was developed (see Multimedia Appendix 1), which is described as an electronic medical record plugin that could exist within the current physician workflow. The wireframe contains three main components aimed to support user needs. The “Past Patients” page allows physicians to see an automated list of patients they have previously cared for alongside data points that focus on postcare assessment and team comparisons. The “Collaboration” page allows users to speak to other physicians who may have cared for the patient, providing the opportunity for the user to ask follow-up questions or explore other inquiries. The “Learning” page allows users to find information in one consolidated place, with an additional option to take notes so information can be easily revisited.

#### Past Patients

Participants endorsed the inclusion of information about readmissions, additionally asking that time to readmission (eg, less than or greater than 30 days) and the readmission diagnosis be included. Participants appreciated the idea that this could be created automatically and reiterated frustrations that they are currently doing much of this work manually. Participants felt strongly about the comparative statistics as being interesting and something they would want to see, although they acknowledged there would be caveats that could influence the accuracy of these numbers.

Most participants felt positive about diagnosis but brought up a concern about trusting the diagnosis chosen, as this item can be inaccurate or a patient can have many diagnoses. Participants thought that the ability to filter through past patients would be helpful for educational purposes when looking for an example of a specific kind of patient, but that they would not likely use it in their clinical workflow.

Participants found the most recent physician and length of stay data to be the least helpful. They showed preference for finding the person themselves in the chart and only having interest in that person if it was someone they knew. For length of stay, they felt that the data would be more prone to uncontrollable variability, making the measure difficult to use.

Most participants agreed that the name, age, diagnosis, and discharge date would be their top data points for finding a past patient. Participants thought that mortality data could be added to the page.

#### Collaboration

Participants generally expressed that they would be unlikely to actively use the page, citing already having too many existing methods with which to communicate with other providers, a dislike of the idea of going into the medical record during off-service time to message others, and again reiterating a discomfort from earlier interviews about reaching out to others that they are less familiar with. They also worried about the potential for this chat to be monitored or used against them in some way later on.

Some participants’ comments spoke to potential benefits such as having a safe, encrypted method of messaging, and asking if they would be able to attach a patient chart or specific note to a message. This would allow them to more accurately ask questions while maintaining appropriate security.

#### Learning

Participants expressed appreciation of having information combined into a single interface, and especially liked that they could have the information side by side with their note-writing interface, allowing them to more directly cite and annotate work notes. Although not always specifically directed toward the note-taking feature, they all expressed interest in being able to somehow note, flag, or “save for later” articles they come across. Participants expressed mixed feelings about the potential for an algorithm to automatically update feeds to match current patient issues, citing skepticism that it would be able to show them what they were really looking for. Instead, they endorsed having this page as a place to check when needing new information or passively flip through in less busy moments. Participants expressed concern that because of integration into the electronic medical record, this would not be easily accessible when not at work. The concern was rooted in the idea that coming across an article or idea can happen at any moment and would ideally be put into a readily accessible repository, which may not be the case for the wireframe.

## Discussion

### Principal Findings

In relation to previous self-assessment models developed in the clinical setting [7,30], our findings are consistent in illustrating that hospitalist clinicians rarely receive formal, regular, and structured feedback about their performance. However, in contrast to these prior models, our findings show that hospitalist physicians spend greater amounts of time specifically using the electronic medical record to perform self-assessment and are less reliant on peers, patients, and structural educational programs. Because the electronic medical record contains the most data available in a single place, is readily accessible to clinicians, and is a familiar and trusted platform, it is an optimal medium to explore for providing feedback to clinicians and supporting their efforts of self-assessment.

The electronic medical record is a resource easily available to hospitalists almost anywhere, providing a large array of information to use to reflect on their work. Even so, we found that the current implementation of the electronic medical record does not quickly facilitate the self-assessment and learning desired by clinicians. The electronic medical record is built for real-time care as opposed to retrospective use. It does not present summative or aggregate data, making it hard to facilitate longitudinal learning or draw conclusions based on multiple cases. We found that clinicians use time-intensive workarounds to assess themselves but often give up on these. In the evaluation of our proposed app, we found that reviewing past patients can be triggered by certain objective markers such as readmission or mortality, or subjective markers such as personal connections to certain patients or curiosity about clinically challenging cases. Given that established models and tools for peer-to-peer feedback, audit and feedback, and patient feedback exist, there is an opportunity for an app to facilitate electronic medical record–mediated self-assessment to improve the quality and standardization of this assessment [13,37,38]. Interviews of experience with our wireframe illustrate that there are some simple features that could quickly provide a strong foundation for clinician self-assessment, such as supporting a baseline database of past patients for a physician to review and organize. Although other main pages in the wireframe, such as Collaboration and Learning, led to interesting discussions, discerning clear value from these will need further exploration.

In considering whether or not to develop this kind of app, institutions should consider how clinicians are already actively looking for this information and creating their own inefficient workarounds. Developing a growth mindset culture or other positive culture around feedback is a key foundation to any intervention to combat the interpretation of feedback more negatively than it is intended [16]. Clinicians should do their best to not feel isolated by their challenges in self-assessment. They can see their workarounds and added efforts as an opportunity to work with technologists and institutions to build solutions. Technologists are challenged to find improved ways to represent this information and combat the generally negative perceptions, highlighting information that can lead to learning points. Positively, the information that clinicians have the most interest in should be easily accessible in the existing data collected. Multimedia Appendix 2 provides more detailed recommendations for institutions, clinicians, and technologists in developing this app in relation to our themes and subthemes.

We believe this is an optimal time for such an intervention. Some health care systems have embraced the learning health system model and have found ways to use the electronic medical record as a key tool in this practice, but challenges remain as the number of possible interventions is immense [39-41]. We believe that this app could engage more users to be aware of their performance and help them make more effective adjustments on their own, as opposed to relying on system-wide changes. With changes in interoperability standards and the proliferation of apps developed to integrate with electronic medical records, creation of such a tool is more technically reasonable than in the past [42,43]. We believe that following an iterative co-design approach or participatory design approach will help lead to the best outcomes for this app [44,45].

### Future Directions

Initial user research has highlighted the electronic medical record as a promising starting point for a feedback platform, and we hope to build on our current work to explore this potential. Although the medical record is central to current users, other approaches with mobile devices, apps, or other technologies could add valuable dimensions not yet captured in our current work. These other technologies will face the challenges of security, interoperability, and data accessibility to a greater degree than the electronic medical record. Applying and adapting frameworks based on behaviors such as metacognitive awareness that were demonstrated by clinicians in these interviews could also help create a more useful platform [46,47]. We hope that starting with a solid foundation of well-liked features can quickly become a branching point for new ideas and areas of focus.

### Limitations

This work comes with certain limitations. Although almost all participants had worked at more than one institution in their careers, all of the participants were currently working in the same large, urban hospital system. This may lead to additional nuances that would be relevant in a more generalized population that were not revealed in this population. Participants skewed toward being less experienced as attending physicians, which may result in a bias in perspectives on feedback. Although newer clinicians may be more actively looking for feedback, more experienced clinicians may have different perspectives that were not fully captured in this work. Future work could further explore cultural differences across institutions and clinicians.

### Conclusion

Our work identifies gaps and challenges in the current feedback and learning systems of hospitalist physicians. We used a qualitative approach to interview and extract themes relevant to the feedback and self-assessment of hospitalist physicians. Based on such information, we outline a gap in current apps and provide recommendations for institutions, clinicians, and technologists on how they could approach building an app to facilitate self-assessment and feedback.

## Appendix

Multimedia Appendix 1 Wireframe developed for second-round interviews and descriptions of each page within the wireframe.

Multimedia Appendix 2 Relevant takeaway items for each theme and subtheme, directed toward institutions, clinicians, and technologists.
