# Supplementary material for: Role of Technology in Self-Assessment and Feedback Among Hospitalist Physicians: Semistructured Interviews and Thematic Analysis
Source: J Med Internet Res. 2020 Nov 3;22(11):e23299. doi: 10.2196/23299 (PMC7671832; doi:10.2196/23299)
Supplement: Multimedia Appendix 1 [file jmir_v22i11e23299_app1.docx]

**Appendix 1:** Wireframe developed for second interviews and descriptions of each page within the wireframe.


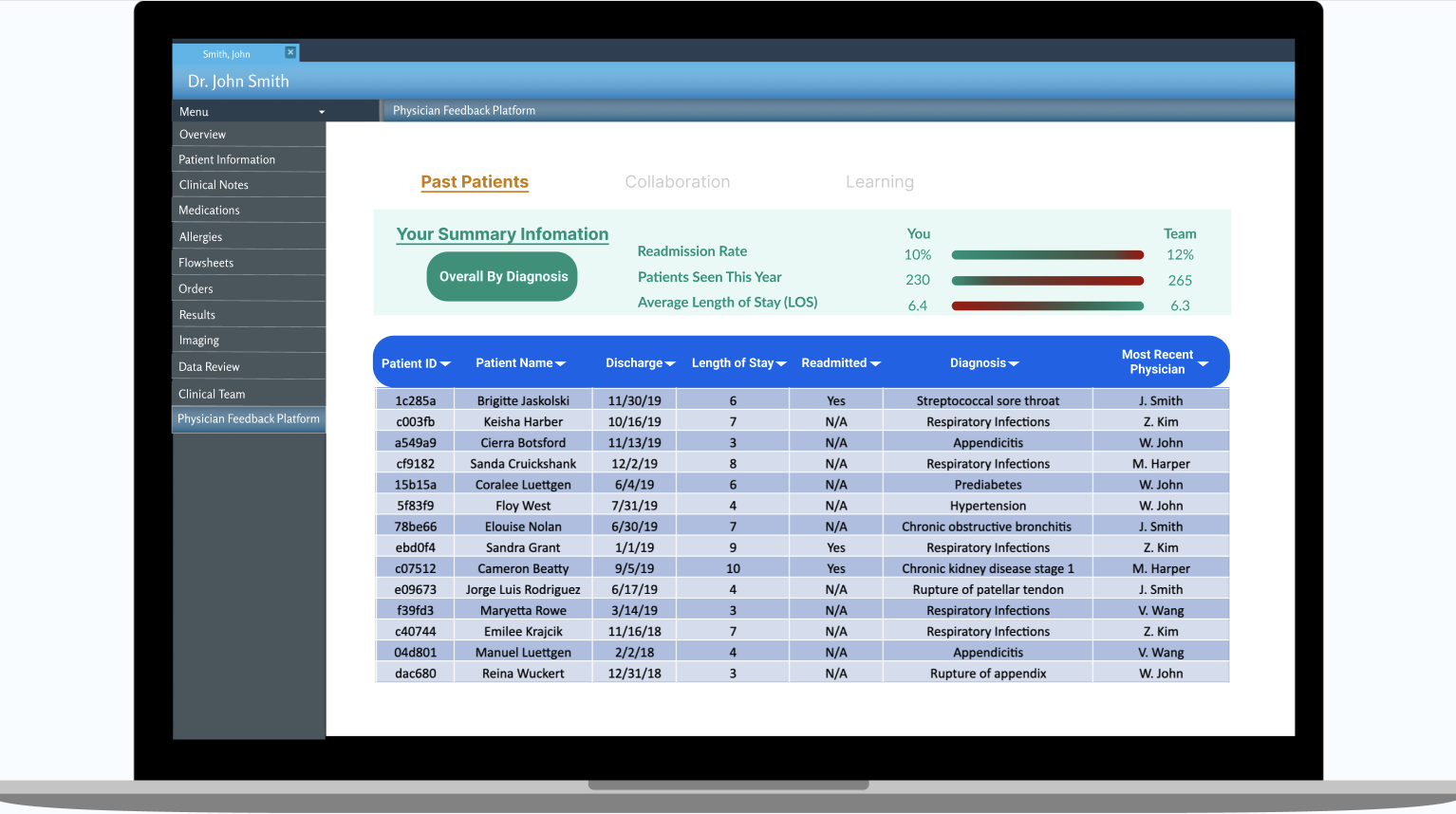


Figure 1: Past Patients page. Physicians can view specific information about their past patients including the date of discharge, length of stay, readmission status, diagnosis, and most recent physician. At the top, the physician can view their performance compared to that of their peers. Physicians can filter by a specific diagnosis or sort the information by another column variable.


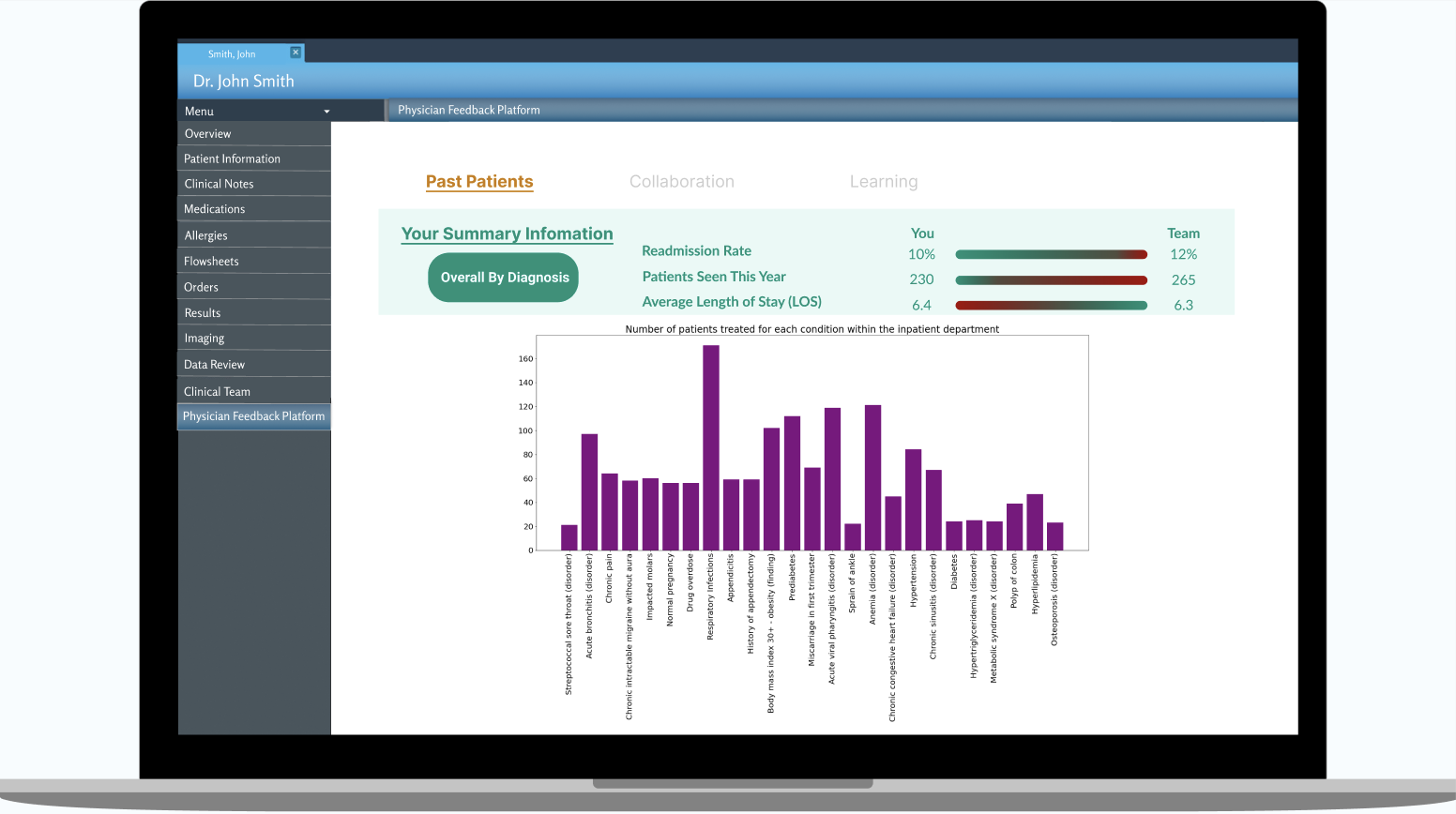


Figure 2: Overall by Diagnosis page within the Past Patient page. Physicians can visualize the relative number of patients with any given diagnosis that they have managed in the past, allowing them to quickly identify patient diagnoses that they have seen often or barely at all.


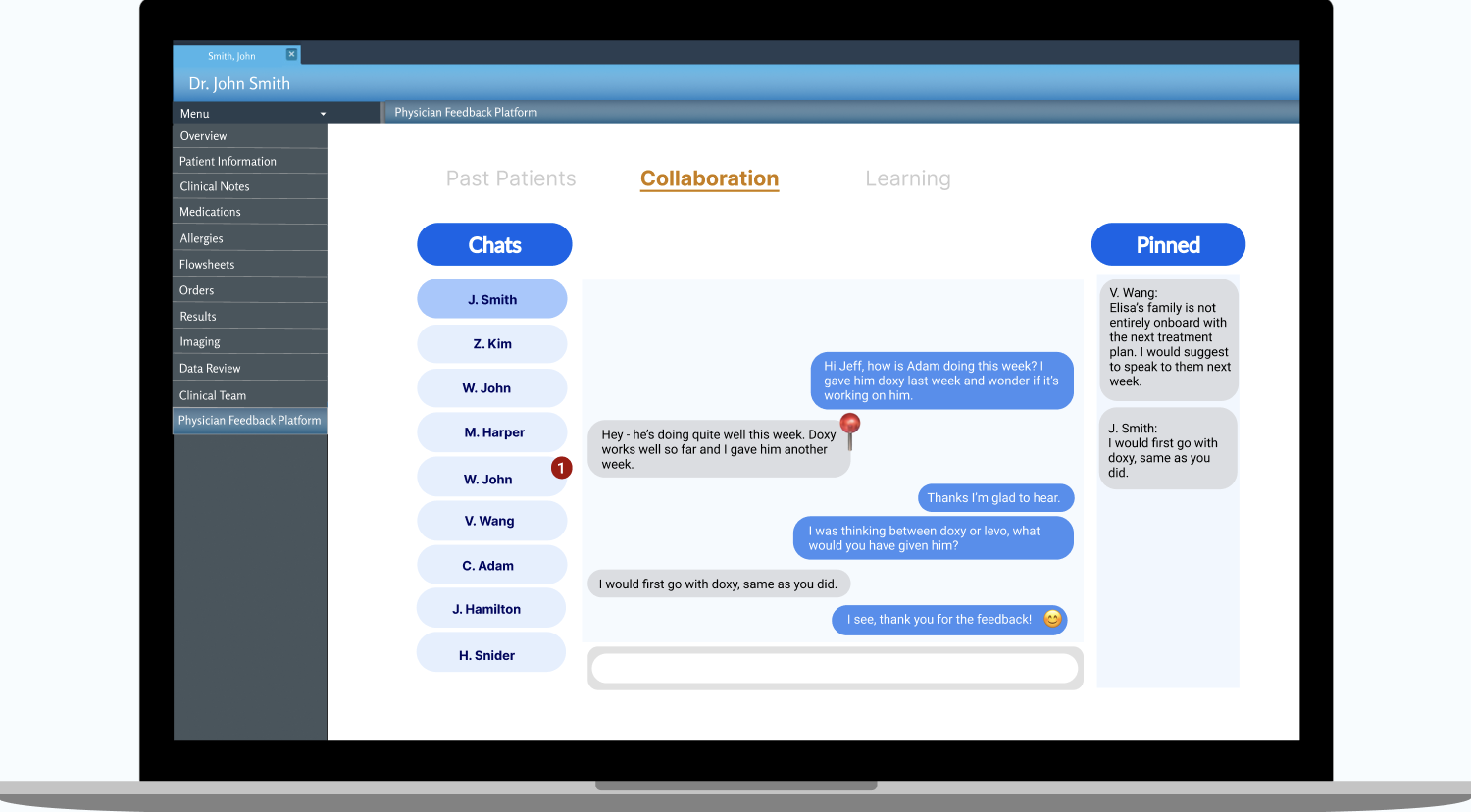


Figure 3: Collaboration page. Physicians can quickly chat with colleagues who have assumed the care of their previous patients, allowing them to follow up on questions or concerns they have. An additional feature allows the clinicians to pin specific comments and save them.


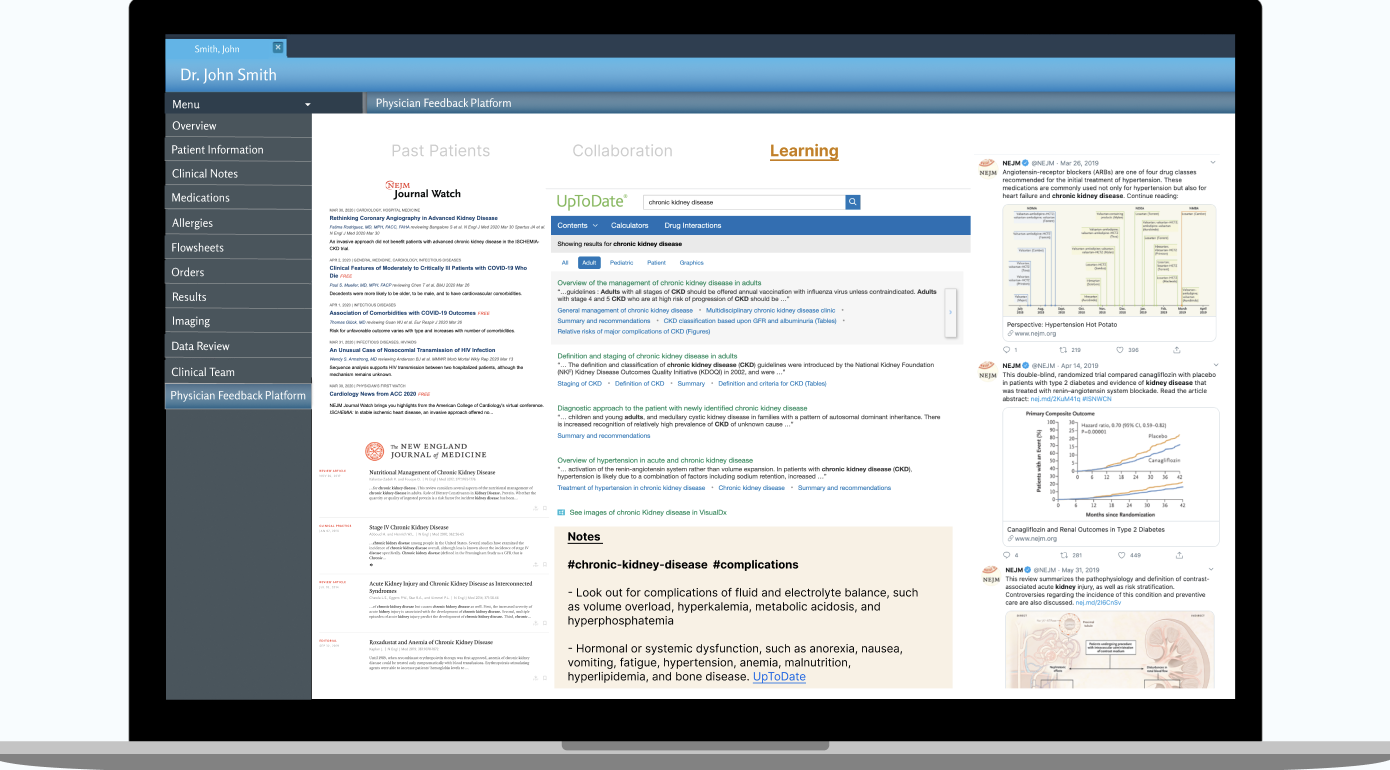


Figure 4: Learning page. Physicians can quickly review information from their favorite journals, UpToDate, or Twitter accounts to find the information they need as questions come up during the care of a patient or after. A notepad allows them to keep track of information of interest as well as save links to relevant papers or articles so that they can find them again later on. Users are able to classify information in the notepad by adding self-defined hashtags.
