# Supplementary material for: Role of Technology in Self-Assessment and Feedback Among Hospitalist Physicians: Semistructured Interviews and Thematic Analysis
Source: J Med Internet Res. 2020 Nov 3;22(11):e23299. doi: 10.2196/23299 (PMC7671832; doi:10.2196/23299)
Supplement: Multimedia Appendix 2 [file jmir_v22i11e23299_app2.docx]

**Appendix 2:**

This table summarizes relevant takeaway items for each theme and subtheme, directed towards institutions, clinicians, and technologists. The recommendations are made to support any of these groups in pursuing a feedback application for clinician growth and can be important starting principles in any discussions about creation of a new technology.

| Themes and  Subthemes | | Institution | Clinician | Technologist |
| --- | --- | --- | --- | --- |
| **Theme I: Collaboration** | |  | | |
|  | I.I: **Physicians are looking for feedback** | Ask what data your physicians use and want otherwise they will try to find it on their own and may come to less informed conclusions. | It is normal to seek this feedback but be aware that it may be misleading or that the data is not currently structured for you to review retrospectively and analyze objectively. | Technology must close the feedback loop with field-specific privacy/regulation considerations. Much of the data that clinicians are looking for already exists in the medical record but is not formatted for review. |
|  | I.II: **Physicians have specific people they consult for feedback** | Building relationships across all team members could support the inclusion of more perspectives and experiences. | Going outside normal circles can expand one’s perspective. Participants express an overwhelming willingness to give feedback if they are asked for it but are otherwise reticent and will lean towards saying nothing. | Technology could augment these existing relationships by allowing attachment of more information in secure ways and could also facilitate random connections to other people. |
|  | I.III: **Physicians interpret feedback more negatively than likely intended** | Due to this tendency, setting expectations and a growth mindset culture becomes more important than ever among teams. | Feedback should not be shied away from due to the negative feelings that it may elicit. In most cases, it is likely that the negative feelings are more related to insecurities/fears than they are the feedback itself. | Clinicians will likely enter with a negative outlook on the data being presented. It is important to explore potential data points that may be more highly valued and more fairly accessed. |
| **Theme II: Self-Reliance** | |  | | |
|  | II.I: **Physicians have go-to resources for learning** | Due to their strong and consistent preferences for certain resources, optimizing or organizing workflows around these could be beneficial without creating more work. | Many resources provide excellent general information (e.g. UpToDate, Journal Club, etc.) but remember that reviewing past patients in the medical record is not structured for retrospective review. | Although combining resources will always sound favorable, it can be equally beneficial to imagine the array of applications in the workflow of the physicians such that the work feels more coordinated and less sporadic amongst these different resources. The clinical day to day remains unstructured in many ways and could benefit from supportive structural innovations. |
|  | II.II: **Physicians build workarounds** | These workarounds are opportunities to improve the systems in use. Increasing customization in platforms or regularly capturing these workarounds can inform future iterations. | When creating these workarounds, be willing to engage with IT teams or electronic medical record managers as these can be potential new features. | Workarounds are valuable insights into features that could be developed. Physicians can be hesitant to share certain workarounds and are uncertain as to what would constitute a workaround. |
|  | II.III: **Medicine can feel like a solo sport** | At times, hospitalists feel obligated to work alone. Due to insecurities about their own knowledge base or lack of structure in the systems they carry many burdens on their own. | There is potential to use this sentiment in order to strengthen relationships with colleagues and recognize that although many decisions are being made alone, many others are making similar decisions. | It is challenging, but building infrastructure to reinforce camaraderie and collaboration can mitigate some of the lone warrior sentiment experienced. |
| **Theme III: Uncertainty** | |  | | |
|  | III.I: **Physicians like numbers but need more context** | Clinicians are skeptical about data points provided due to the vast variation that can occur, this data should be used as a guidepost but without context should not be used as a hard line in the sand. | Although this issue occurs in many data points, solely relying on individual cases without summative data can equally lead to biased conclusions and take-aways. | Data visualization in a way that can tell more about each data point than just a number could significantly improve a clinician’s assessment process. e.g. readmission color coded by time window and indication of diagnosis. |
|  | III.II: **Physicians cannot always find the “right” answer** | In many cases, the takeaway remains unclear. It is not feasible to simply delineate whether or not every decision was correct or not, institutions could embrace and acknowledge this uncertainty. | Uncertainty is a large part of the clinical process, expressing and discussing the uncertainty with peers or others could be beneficial when these answers are not straight forward. | Although data will be used for reflection, it may not always have a right answer, thus data representation should focus on clarity of the information rather than expecting that each data point would necessitate a change. |
|  | III.III: **Physicians’ actions are limited by uncertainty about workplace cultural expectations** | Clinician collaboration and search for feedback are strongly influenced by the workplace expectations they perceive. | Although expectations may not be well defined, feedback should not be made more daunting than it is meant to be. Be unafraid to share and discuss uncertainty. | Provide alternative ways to close the gaps in in-person communication, and aim to deescalate perceived cultures around feedback. |
